# The State of the Journal

**DOI:** 10.3402/ijch.v74.27617

**Published:** 2015-03-02

**Authors:** Kue Young

It is that time of the year again to provide our readers with a progress report on the journal's health and well-being, and to provide a “sneak preview” of the coming months.

The journal's editorial staff underwent some changes during 2014. Kue Young continues as editor-in-chief, with 3 deputy editors: Marit Jørgensen (Denmark), Rhonda Johnson (United States) and Anders Koch (Denmark). The chief and deputy editors oversee the management of manuscripts. They were ably assisted by 3 associate editors: Juhani Leppäluoto (Finland), Jon Øyvind Odland (Norway) and Pamela Orr (Canada), who have committed to reviewing manuscripts that are in their areas of expertise.

Between January and December 2014, 76 new manuscripts were submitted to the journal website, an average of about 1 new submission every 5 days. The total for 2014 was an increase over 2013, when 60 manuscripts were submitted.

The acceptance rate continues to fall, and has fallen below 50% among the “cohort” of manuscripts submitted up to December 31, 2014, compared to 60% among manuscripts submitted in 2013, and much higher in earlier years.

Authors are justifiably concerned about the time they have to wait before they see their scientific (and literary) labours bear fruit. For the 29 papers *published* in 2014 (excluding editorials, book notices and the special issue on Yakutia), the time elapsed from receipt to acceptance was 71 days, and the average time from receipt to publication was 105 days. It can be seen that we have made improvement in reducing the time a manuscript spends in the review and production processes. Here I want to make a plea to our authors and readers. In order for the waiting time to be further shortened, we need reviewers! The task of finding willing reviewers is getting more difficult. When asked, many potential reviewers claim lack of time, while some do not even bother to respond.

What are we planning for the journal in 2015? We welcome submissions in all the categories of manuscripts. We shall start a new category, called Theory and Methods, to cater to articles that deal with theoretical issues in research or policy to stimulate discussion. We also welcome articles that describe their theoretical framework and provide details of the design of complex studies, as generally articles reporting results of original research tend not to have sufficient space for such information.

We shall have a special issue on suicide in circumpolar regions, especially among Indigenous youths. The journal published such a special issue in 2009. Alas, there is little sign that the magnitude of this serious health problem has diminished.

We thank all our readers, authors and reviewers, without whose support, this journal will be an empty space. The editorial and production staff at Co-Action Publishing continues to demonstrate their high degree of professionalism. Last but not least, the website has undergone a major facelift, and now there is a fresh new look consistent across all journals published by Co-Action Publishing.

*Kue Young* Editor-in-Chief Email: kue.young@utoronto.ca

